# Sexual Stimuli Cause Behavioral Disinhibition in Both Men and Women, but Even More So in Men

**DOI:** 10.1007/s10508-022-02514-1

**Published:** 2023-01-24

**Authors:** Julian Wiemer, Steffen Kurstak, Florian Sellmann, Kerstin Lindner

**Affiliations:** 1 Department of Psychology, Institute of Psychology (Biological Psychology, Clinical Psychology, and Psychotherapy), University of Würzburg, Marcusstr. 9-11, 97070 Würzburg, Germany; 2 Psychotherapy Center for Sexual and Violent Offenders Würzburg, Würzburg, Germany

**Keywords:** Response inhibition, Sexual stimuli, Emotion, Sex differences, Go/No-Go Task

## Abstract

In our society men are considered more impulsive than women, especially in the violent and sexual domain. This correlation of sex and impulsivity might trace back to enhanced male impulsivity in general or a domain specific effect of emotions on impulsivity. The evidence for sex differences in the interaction of emotional or sexual stimuli and impulsivity has been relatively inconclusive so far. In this study, we investigated the effects of various emotional stimuli on responsivity in a Go/No-Go task. Participants had to respond quickly to a visual cue and withhold their response to another visual cue, while different emotional pictures were presented in the background, including sexual stimuli, non-sexual positive stimuli and negative stimuli. Both men (*N* = 37) and women (*N* = 38) made most commission errors in the sexual condition, indicating a disinhibiting effect in both genders. On top of this, men made even more commission errors than women, specifically in the sexual condition and not in other conditions. Men rated sexual stimuli as more positive, but did not differ from women in arousal ratings and pupil dilation. These findings may partly indicate increased impulsive behavior under sexual arousal in men, most likely driven by enhanced approach motivation due to more positive value but not higher arousal of sexual stimuli. The results are consistent with the theory of evolutionarily based concealment of sexual interest in women.

## Introduction

Emotions can be conceptualized as strongly associated with action tendencies (Frijda, 1987). When we are happy, we are more likely to move, to go out and toward people, while sadness can lead to withdrawal. Likewise, sexual arousal prepares an organism for sexual activity and ultimately reproduction. Thinking about sex or seeing sexual cues leads to the release of sex hormones such as testosterone in men and women, which in turn modulates genital function and the processing of social stimuli (Bancroft, 2005; Hellhammer et al., 1985; van Anders & Watson, 2006; van Honk et al., 2005). Physiological arousal includes changes in genital pulse amplitude, skin conductance and pupil dilation (Attard-Johnson et al., 2021; Costa & Esteves, 2008; Heiman, 1977). Motivational models assume the initiation of a state of wanting (Berridge & Robinson, 2016; Georgiadis & Kringelbach, 2012). In the brain, activity is enhanced, among other regions, in visual processing areas and premotor cortex, which is involved in action imitation (Georgiadis & Kringelbach, 2012). In fact, both men and women are more likely to engage in sexual activity after watching sexually arousing videos (Both et al., 2004). Even subliminal sexual stimuli have the potential to facilitate genital responses and activity in motivational brain areas (Ponseti & Bosinski, 2010; Wernicke et al., 2017). Thus, sexual stimuli can trigger a strong action tendency in the human body and mind, but how strong is this action tendency and how easy can it be controlled when action execution is not indicated?

Sexual arousal might also end up in regretful actions, such as unprotected sex, infidelity or transgressive sexual behavior. Men and women estimate their own sexual disinhibition as more likely when they are in a state of sexual arousal (Imhoff & Schmidt, 2014) and men transgress boundaries in an imaginary date script more if they are sexually aroused (Spokes et al., 2014). Although findings in laboratory paradigms like these should be generalized to everyday behavior with care, they might help us understand the interaction between sensory stimulation, sexual arousal and strong action tendencies that can be difficult to control. 

Regarding the violation of social norms in general, there are remarkable differences between women and men. Men are held responsible for the most part of violent (79%) and sexual offences (91%), especially in severe cases (German Federal Crime Police Office, 2020). Also, pornography addiction rates seem to be higher in men (Rissel et al., 2017). The reasons for these asymmetries may be complex and multi-causal, but one reason might be stronger (or less regulated) action tendencies triggered by emotional states. 

In accordance with this assumption, men are often considered as more impulsive than women. While impulsivity in general can be described as a predisposition to fast and unplanned reactions to stimuli without considering potential negative consequences (Moeller et al., 2001), several subcomponents have been identified that can be widely attributed to increased approach motivation, deficient inhibitory control and higher order decision based impulsivity (Cross et al., 2011). Having a closer look at subcomponents of impulsivity, a meta-analysis came to the conclusions that men show higher sensation seeking and risk-taking, as well as lower punishment sensitivity while no differences in reward sensitivity were observed (Cross et al., 2011). A review focusing on impulsive action and impulsive decision making found increased impulsive action in males depending on the behavioral task (go-nogo rather than stop-signal-tasks), and a tendency toward increased impulsive decision making in females (Weafer & de Wit, 2014). 

The Go/No-Go task is a widespread method to study impulsivity and response conflict in the cognitive sciences (Donders, 1969). In go-nogo tasks, two kinds of stimuli are presented to the participant: a Go stimulus that demands a quick response (e.g., a square) and a noGo stimulus that requires to withhold any reactions (e.g. a circle). Usually, Go trials are more frequent than No-Go trials which is assumed to establish a prepotent tendency to respond. This will make it necessary to withhold this response in the less frequent nogo trials. The number of commission errors in these nogo trials is typically used as an index of failed response inhibition, resp. impulsivity. However, it should be considered that the process of response inhibition is deducted indirectly and it is a matter of debate, if inhibitory processes are in fact necessary to explain the variance in responsiveness. Alternative models conceptualize hyper-responsiveness as the result of a value-based choice that sets off when the value of responding increases, outweighs the value of not responding and crosses a critical threshold (Berkman et al., 2017; Veling et al., 2022). In either case, the go-nogo task enables us to study the potential of different emotional stimuli to cause (hyper-) responsiveness in individuals. Responsivity in go-nogo tasks is associated with different psychopathological states, suggesting that performance in this task can be related to some degree to real-life impulsivity (Wright et al., 2014). In stop-signal tasks, one has to react to a Go stimulus, while sometimes a stop signal is presented in addition to the Go stimulus with a varying delay that is adjusted so the error rate is kept constant, usually at 50%. 

So far, it seems evident that men are more impulsive than women in certain regards, but how does the emotional and motivational context influence impulsivity in men and women? Particularly, are there any sex differences in the emotional modulation of impulsivity? Given the apparent differences outside the laboratory, studies concerning this question are surprisingly rare so far and results are considerably mixed. 

One study found that women committed more errors, i.e., responded more impulsively, in a sexually arousing go-nogo task than men—contrary to the aforementioned differences in real life impulsivity (Macapagal et al., 2011). In this experiment, participants had to respond to specific neutral or sexual images and inhibit their responses to specific other neutral or sexual images. As the authors note, since the key press terminated the presentation of the pictures, men might have been motivated to slow down their responses, thus prolonging viewing time and reducing error rates. Another study then found the opposite effect in a stop-signal-task using erotic images and painful video clips (Yu et al., 2012). Men needed a longer stop response time than women in both sexual and painful conditions, indicating stronger response inhibition in women. Women did not show any differences between emotional and neutral conditions. Finally, a third study investigating the emotional modulation of response inhibition in men and women, neither did find sex differences nor any effects of the emotional context on task accuracy, but did find sex differences in electrophysiological potentials, i.e., women’s amplitude of the inhibition related N2 component was enhanced (Ramos-Loyo et al., 2016). 

Besides sex differences, the findings of the emotional modulation of response inhibition in general have also been ambiguous so far. One study found more commission errors for (especially negative) high arousing stimuli than low arousing stimuli (de Houwer & Tibboel, 2010). Another study did not find deviant error rates for negative stimuli, but increased Go response times (Littman & Takács, 2017). The increased response times for negative images may depend on the specific content of images, as one study found this to be true only for mutilations while threatening images rather speeded up response times (Buodo et al., 2017). Only positive stimuli led to an increase in commission errors in this study. 

We assume that methodological differences contribute to these heterogeneous findings. For example, in some studies the presentation of pictures was terminated with a button press. In this case, participants might be attracted by a positive image and feel an impulse to press the button as in an approach behavior, but at the same time they might be motivated to keep looking at the picture which may counteract the approach behavior. In addition, sometimes emotional images were simultaneously used as motivational context and as the Go or No-Go stimuli themselves. Hence, response times might be significantly affected by identification and memory processes. Finally, the timing between an emotional stimulus and a Go stimulus might be crucial, as one study suggests (Contreras et al., 2013). These experiments showed that the typically expected pattern of shortened response times for positive stimuli and prolonged response times for negative stimuli was only observed, when the stimulus onset asynchrony between emotional picture and Go stimulus was 200 ms but not when it was 600 ms. 

Consequently, in order to make a contribution to the literature of sex differences in the emotional modulation of responsivity, we designed a go-nogo experiment considering the following methodological parameters. First, picture duration was fixed, irrespective of button press, thus viewing time did not interfere with approach motivation. Second, emotional pictures and go stimuli were separate visual cues. In order to further minimize the task relevance of the emotional pictures, they were presented in black and white in the background of a blue Go stimulus. Third, we purposefully set the time gap between emotional stimulus and go (or nogo) stimulus at 200 ms. Fourth, we included neutral, sexual, negative and positive emotional pictures to test the specificity of erotic stimuli and compare effects with non-sexual positive emotions. Finally, pupil dilation was assessed throughout the experiment as an indicator of autonomous arousal. The dilating and constricting muscles of the pupil are innerved by sympathetic and parasympathetic control (Larsen & Waters, 2018). Typically, positive and negative arousing emotional pictures evoke a dilation of the pupil (Bradley et al., 2008). We included this measure to validate emotional responsivity on a more objective, physiological level. Previous studies also showed that the pupil can index sexual arousal and, in men, possibly sexual orientation (Attard-Johnson et al., 2021). 

On the basis of the above-mentioned sex differences in impulsivity, we expected men to display more commission errors in nogo trials and/or shorter response times in Go trials than women, if sexual or negative stimuli are present. As negative stimuli we used pictures of ill or injured people to provoke an inhibiting effect. If such an inhibitory effect contributes to sex differences in violent acts, men should show reduced response inhibition. 

## Method

### Participants

Participants were recruited via advertisements on local boards and websites such as the University’s online registration system for psychological studies. This study is part of a larger project involving violent and sexual offenders who also participated in the response inhibition task, but were not included in the present sample. Originally, 107 participants were recruited as control participants besides offenders. We excluded 1 man because he had been convicted of assault previously. For the purpose of matching age to the offender group, the original sample contained more older men than women. In addition, performance and response time typically decline with age (Votruba & Langenecker, 2013), so we excluded 15 participants above the age of 50 years and the 5 oldest men to match both age and sample size to women. Further, 5 indicated to be homosexual and 1 did not disclose sexual orientation. Since sexual images used in this study depicted heterosexual activities, we only included individuals who indicated to be heterosexual or bisexual. In addition, 2 participants did not complete the task, 2 made more than 50% commission errors and 1 made an unusual amount of omission errors (98% vs. average 2%), suggesting they failed to follow the instructions. Overall, these exclusion decisions did not substantially change the significance of the main results, i.e., the interaction between emotion and sex in commission errors (no exclusions: *p* = .046, without age matching: *p* = .024, including all sexual orientations: *p* = .001, incl. more than 50% commission errors: *p* = .014, incl. non-completers: *p* = .005). The final sample included 37 men and 38 women. The sample size was chosen based on a power analysis to find a medium sized main effect within each group and a within-between group interaction with a statistical power of at least 0.80. A power analysis using G*Power (Faul et al., 2009) and assuming a correlation of *r* = .50 and a non-sphericity correction of = .50 suggested a sample size of 38 per group. The finally achieved power to detect a significant interaction considering final values was 0.99 for commission errors and 0.91 for response times.

### Materials

#### Emotional Pictures

We used 48 emotional pictures to induce emotions during the Go/No-Go task, 12 for each of the four categories neutral, positive, negative, and sexual. The pictures were derived from different collections specifically intended for research purposes (IAPS; Lang et al., 2008; EmoPics, Wessa et al., 2010; OASIS; Kurdi et al., 2017), supplemented by images from web searches. This wide pool of images enabled us to select stimuli depicting humans in comparably complex scenes and in a particularly arousing way. Specifically, sexual images were intended to be arousing and ecologically valid for typical pornographic images. Thus, sexual stimuli depicted a male and a female during sexual intercourse, while the whole bodies of both persons were visible. Neutral pictures showed people during low arousing activities like office work, chess play, reading or a walk. Positive stimuli depicted happy people in high arousing scenes such as sledding in the snow, riding a carousel or cheering in sports. Negative pictures showed bodies of severely injured, ill or emaciated individuals. All pictures were gray-scaled and adjusted regarding size (800 × 600 pixels), luminance and contrast with the help of picture editing software.

#### Go/No-Go Task

**Fig. 1:**
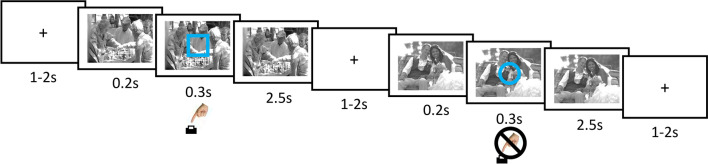
Trial sequence of a go and a nogo trial in the Go/No-Go task. A blue square or circle was superimposed on grayscale emotional pictures for 300 ms with a stimulus onset asynchrony of 200 ms. Participants were to respond as quickly as possible to the Go stimulus (a square in the illustrated example; counterbalanced across participants) and to withhold responses to nogo stimuli (a circle here). As a reference they should try to respond within the presentation time of the blue Go stimulus. At they same time, they were asked to make as few errors as possible. The inter-trial-interval was randomized between 1 and 2 s. Go response times were calculated for valid responses between 150 and 1000 ms following the Go stimulus. Depicted are not the original pictures used in the study, but similar ones, to prevent public access and preserve copyrights

In the go-nogo task, participants were asked to respond by button press as quickly and accurate as possible to a blue square (or circle) superimposed on one of the emotional pictures in every trial (see procedure for details and Fig. 1 for an illustration). Critically, participants are not allowed to respond to the other type of stimulus (circle or square). If they respond anyway, these reactions are counted as commission errors and can be interpreted as a sign of response selection or failed inhibitory control (Wessel, 2018). Commission errors and reaction times are the main outcome variables of the go-nogo paradigm. Reaction times in Go trials have been interpreted as a response bias, an approach tendency, vigilance, decision making, response initiation, and preparedness (Meule, 2017; Wright et al., 2014). We calculated odd–even split half reliability of these measures in the present sample. They were acceptable for commission errors (neutral: *r* = .73, positive: *r* = .78, negative: *r* = .69, sexual: *r* = .75) and excellent for reaction times (neutral: *r* = .97, positive: *r* = .97, negative: *r* = .98, sexual: *r* = .97). They are also comparable to other assessments of go-nogo reliability (Williams & Kaufmann, 2012). Considering the validity of this task, a meta-analysis showed association between task performance and psychopathological states, such as decreased impulsivity for anxiety disorders and increased impulsivity for bipolar disorder (Wright et al., 2014). Go-nogo tasks do evoke electrophysiological indices of inhibition in the brain, although a high pressure on quick responses seems to be necessary (Wessel, 2018). The relationship with self-reported motoric impulsivity in the BIS-15 was small but significant in the present sample (*r* = .27).

#### Demographic Data

The following demographic data were assessed: Education as the last educational institution graduated (1 = no graduation to 7 = university graduation), handedness (left, right or bimanual), the ovulation phase for women (day 1 = start of menstrual period; days 1–9 = menstrual, days 10–15 = follicular, day 16+  = luteal phase), pornography use and videos containing violence (1 = never, 2 = less than weekly, 3 = several times weekly, 4 = daily), regular alcohol, nicotine, cannabis and other drug use (yes or no).

#### Sexual Experience Survey (SES)

We used a shortened version of the German version of the SES Short Form Perpetrator (Koss et al., 2007) which assesses any former use of physical or verbal enforcement of sexual interactions (including kissing), or the exploitation of intoxicated partners on a four-point-scale (9 items, 1 = no, 4 = three times or more). 

#### Aggression Questionnaire (AQ)

Aggressive personality traits were measured with the short version of the German version of the AQ (Buss & Perry, 1992; von Collani & Werner, 2005). It includes four dimensions of aggression: physical and verbal behavior, anger as affect and mistrust as a cognitive component. Participants rate the fitting of experiences and traits to their personality on a four-point-scale (12 items, 1 = not at all, 4 = completely).

#### Barratt Impulsiveness Scale (BIS-15)

Impulsivity as a personality trait was measured with the BIS-15 (Meule et al., 2011; Spinella, 2007). It includes three dimensions of impulsivity: non-planning, motor impulsivity and attentional impulsivity. Ratings on a four-point-scale relate to the frequency of different behavior (15 items, 1 = never/rarely, 4 = almost always/always). 

#### Emotion Regulation Questionnaire (ERQ)

Emotion regulation style was assessed with the ERQ (Abler & Kessler, 2009; Gross & John, 2003) which contains two subscales, reappraisal and suppression. Items refer to regulation strategies for positive and negative emotions (10 items; 1 = not at all true, 7 = completely true).

### Procedure

The experiment took place in a dimly lit room using a 14″ Notebook (1680 × 1050 pixels resolution) and lasted about one hour. At the beginning, participants signed formed consent and filled out questionnaires about demographic data, the SES, the AQ, the BIS-15, and the ERQ. Then, the eye tracker was calibrated, the luminance of the room was measured with a luminance meter and the experimental tasks started.

#### Go/No-Go Task

The task was set up with Presentation (Neurobehavioral Systems, Berkely, CA, USA). Participants were instructed on screen to respond by pressing the space bar whenever they see a blue square (or circle; Go stimulus) and not to respond to the other stimulus (noGo stimulus). They were asked to respond as quickly as possible and at the same time to try to avoid any errors, but that it is more important to be quick than to make no mistakes. As a benchmark, we asked them to try to respond within the time the blue square (or circle) was visible (300 ms). Participants were randomly assigned to one of the two versions (square = go and circle = nogo; circle = go and square = nogo). They were also informed that pleasant and unpleasant pictures were presented simultaneously and that they may abort the experiment in case of discomfort. Each trial started with a fixation cross in the middle of the screen, lasting randomly between 1 and 2 s. Then, the emotional picture was presented alone for 200 ms. After 200 ms, the go or noGo stimulus was superimposed on the picture for 300 ms (see Fig. 1). Then the emotional picture was presented alone for 2500 ms. Before the actual experiment started, participants absolved a training session with 15 trials in which five neutral pictures were used that did not occur later in the experiment. In this training session but not later, they received feedback onscreen in case of an error ("Error!") or if the response time exceeded 400 ms ("Faster!") after picture presentation for 500 ms. After checking that the participants had understood the task, they started with the emotional go-nogo task. The task involved 288 trials distributed to three consecutive blocks of 96 trials. Each block contained 16 Go trials and 8 nogo trials for each of the four emotional conditions (neutral, positive, negative, sexual). The response conditions were included in the ratio of 2:1 in order to increase the difficulty of response inhibition. Between blocks and in the middle of each block, a short break was included and participants could continue when they were ready. In addition, they received feedback on their mean response time.

After the go-nogo task was completed, participants were shown each picture again for three seconds and asked to rate valence (0 = very unpleasant, 50 = neutral, 100 = very pleasant) and arousal (0 = calm, 100 = very arousing) on visual analogue scales. 

#### Acquisition of Pupil Dilation

Pupil diameter was measured with a Tobii Pro Nano eye tracker (Tobii Pro, Stockholm, Sweden) at a sampling rate of 60 Hz throughout the go-nogo task and the rating phase. The eye-tracker works from a distance with infrared illumination and was mounted below the screen. Before the experiment started, participants absolved a calibration procedure, in which the distance and angle toward the screen were optimized and detection of fixation points in the corners of the screen were checked. In addition, room luminance was measured with a luminance meter to control for any potential luminance related influences on pupil dilation. Using a MATLAB-based script, pupil diameter was cleaned from artifacts, such as blinks and other rapid changes in diameter. Blinks and artifacts were interpolated via linear interpolation. In addition, the data were low-pass filtered at 10 Hz, and baseline corrected to 500 ms before stimulus onset. Trials with more than two consecutive seconds of interpolated data were discarded from the analysis. On average, 3.1% of trials (max. 27%) in the go-nogo task and 5.3% (max. 40%) of trials in the rating phase were marked as bad by this procedure. One participant was excluded from the rating analysis because more than 50% of trials were marked as bad (73%). In addition, due to technical failures, pupil data were missing from several participants, leaving 34 men and 38 women for the go-nogo task and 33 men and 34 women for ratings.

### Data Analysis

We report how we determined our sample size, all data exclusions, all manipulations, and all measures in the study. The data were analyzed using SPSS Statistics V25.0 (IBM, 2017). Sample characteristics were analyzed with exploratory between group t-tests or chi-square-tests (for categorical variables) without multiple comparison correction to explore for potential differences between groups in terms of demographic and personality traits. Error rates, reaction times, emotion ratings and pupil dilation were analyzed with mixed ANOVAs including dependent variables (emotion, time) and participant sex as an independent variable. For all analyses, *p*-values below an alpha-level of .05 were considered as statistically significant. Follow-up t-tests were conducted for pairwise comparisons and corrected according to Bonferroni-Holm, dividing an alpha of .05 by the number of remaining tests, starting with the lowest *p*-value. *t*-tests were two-sided if not otherwise specified. If sphericity was violated in ANOVAs, we used Greenhouse–Geisser corrected *p*-values. Means are reported ± standard deviations. In addition, the 95% confidence intervals (CI) are reported for difference values.

Commission errors were counted as the percentage of responses in a condition to nogo stimuli that occurred within the presentation time of the emotional picture. Go reaction times were counted as the mean response time to a Go stimulus between 150 and 1000 ms after the occurrence of the Go stimulus (Buodo et al., 2017; De Houwer & Tibboel, 2010). This time window was chosen to exclude premature responses and outliers. Responses outside this window were excluded from the analysis. For illustration purposes, we used raincloud plots based on code by Allen et al. (2021). Data are available at 10.6084/m9.figshare.20237508.

## Results

### Sample Characteristics

Demographic and psychometric sample characteristics can be obtained from Table 1. Men and women did not differ in age, education, handedness and room luminance (which was assessed as a control variable for pupil dilation). Men disclosed more alcohol, nicotine and pornography consumption. No differences were found for watching violence videos, as well as cannabis and other drug taking. Men tended to be more verbally aggressive, while men and women were comparable in terms of sexually intrusive behavior, impulsivity and other aggressive behavior. There was a non-significant trend for more emotion suppression and less reappraisal in men. Women’s stage of the menstrual cycle was equally distributed across the menstrual, follicular and luteal phases.

**Table 1:** Demographic and psychometric sample characteristics

	Women	Men	*p*
Age (in years)	26.03 (5.57)	27.59 (4.33)	.178^t^
Education	6.18 (0.61)	5.97 (0.96)	.525^χ2^
Handedness	38 R, 0 L, 0 B	34 R, 1 L, 2 B	.201^χ2^
Ovulation phase	12 M, 10 F, 11 L	-	–
Room luminance	9.30 (22.35)	5.80 (3.62)	.357^t^
Pornography	1.82 (0.39)	2.27 (0.73)	.003^χ2^
Violence Video	2.08 (0.43)	2.19 (0.57)	.485^χ2^
Alcohol	42%	65%	.048^χ2^
Cannabis	5%	8%	.621^χ2^
Nicotine	8%	32%	.008^χ2^
Other drugs	0%	0%	–
SES	1.03 (0.15)	1.01 (0.06)	.501^t^
AQ physical	1.25 (0.44)	1.36 (0.53)	.346^t^
AQ verbal	1.70 (0.51)	2.00 (0.67)	.033^t^
AQ anger	1.97 (0.67)	1.89 (0.65)	.592^t^
AQ mistrust	1.61 (0.60)	1.75 (0.66)	.329^t^
AQ total score	1.63 (0.38)	1.75 (0.47)	.244^t^
BIS-15 nonplanning	1.94 (0.55)	2.06 (0.61)	.382^t^
BIS-15 motor	2.22 (0.52)	2.18 (0.58)	.802^t^
BIS-15 attention	2.01 (0.54)	2.06 (0.50)	.686^t^
BIS-15 total	2.06 (0.37)	2.10 (0.46)	.643^t^
ERQ reappraisal	5.07 (0.79)	4.62 (1.15)	.053^t^
ERQ suppression	3.30 (1.36)	3.91 (1.44)	.061^t^

Education measured in categorical steps from 1 (no graduation) to 7 (university); handedness: R = right, L = left, B = bimanual; Ovulation phase: M = Menstrual (day 0–9), F = Follicular (10–15), L = Luteal (16 +); room luminance measured in lux; pornography and violence video consumption from 1 (never), 2 (less than weekly), 3 (several times a week) to 4 (daily); SES = Sexual Experiences Survey, AQ = Aggression Questionnaire, BIS-15 = Barratt Impaulsiveness Scale 15, ERQ = Emotion Regulation Questionnaire, t = *t*-test, χ^2^ = chi-square-test

### Commission Errors on No-Go Trials

An ANOVA with the within-subjects factor emotion (neutral, positive, negative, sexual) and the between-subjects factor sex (male, female) revealed a significant large main effect of emotion, *F*(3, 219) = 37.97, *p* < .001, *η*_*p*_^2^ = 0.34, *ε*_GG_ = 0.85, and a significant Emotion X Sex interaction, *F*(3, 219) = 5.36, *p* = .003, *η*_*p*_^2^ = 0.07, *ε*_GG_ = 0.85. The main effect of *sex* was also significant, *F*(1, 73) = 4.15, *p* = .045, *η*_*p*_^2^ = 0.05.

**Fig. 2:**
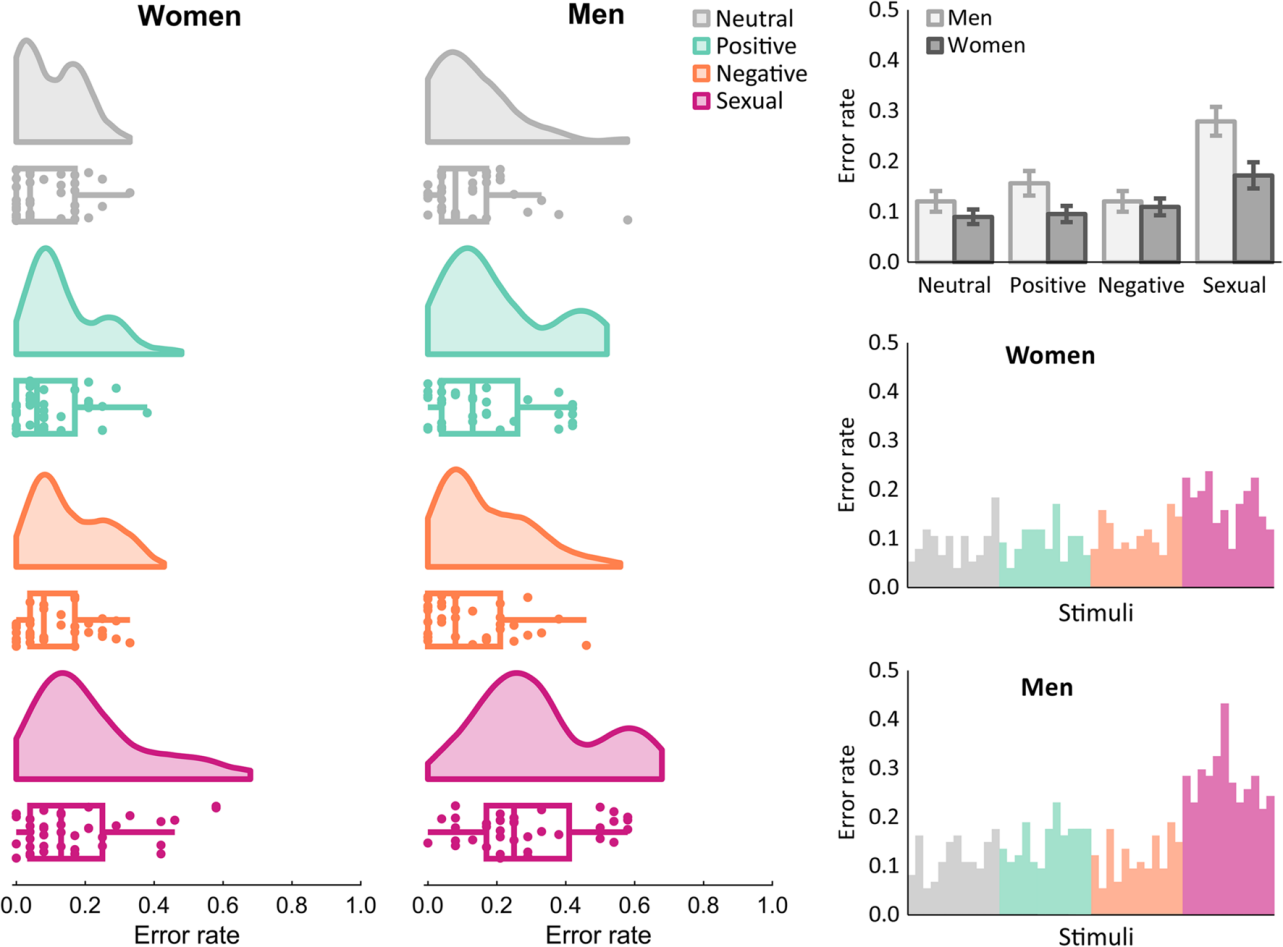
Commission errors to No-Go stimuli. The left side shows raincloud plots including boxplots and the distribution of error rates across participants. As can also be seen in the bar plot with mean values on the top right side, both men and women committed more errors in the sexual condition, while men committed also more errors than women in the sexual condition. Error bars indicate standard errors of the mean. Mean error rates for individual pictures used in the study can be obtained from the two bar plots on the mid and lower right side.

In men and women only sexual stimuli led to an increase in commission errors, compared to the neutral condition, all *p* < .001 (see Table 2). The effect was medium in women, *d* = 0.55, and large in men, *d* = 1.02. This difference in effects was significant, *p* = .016. Also, commission errors during sexual images were more common than in both other emotion conditions, *p*s < .001. While positive stimuli might be considered as trending toward more commission errors relative to neutral stimuli in men (and relative to women), no other significant differences were found. Overall, men tended to commit more errors, but this only kept up for sexual images, *t*(73) = 2.79, *p* = .007, *d* = 0.64. The group difference for positive images was only marginal significant, considering multiple comparisons, *p* = .039 (Fig. 2).

**Table 2:** Statistics of commission errors in No-Go trials

	Men (*N* = 37)		Women (*N* = 38)		
	Mean (SD)	vs. Neutral	Mean (SD)	vs. Neutral	Men vs. Women
Neutral	.12 (.13)	–	.09 (.09)	–	*d* = 0.28, *p* = .227 95CI [−0.02, 0.08]
Positive	.16 (.15)	*d* = 0.26, *p* = .052 95CI [−0.00, 0.07]	.10 (.10)	*d* = 0.06, *p* = .661 95CI [−0.02, 0.03]	*d* = 0.49, *p* = .039 95CI [−0.00, 0.10]
Negative	.12 (.12)	*d* = 0.00, *p* = 1.00 95CI [−0.04, 0.04]	.11 (.10)	*d* = 0.21, *p* = .138 95CI [−0.01, 0.05]	*d* = 0.10, *p* = .679 95CI [−0.04, 0.06]
Sexual	.28 (.17)	*d* = 1.02, *p* < .001^a^ 95CI [0.12, 0.20]	.17 (.16)	*d* = 0.55, *p* < .001^a^ 95CI [0.04, 0.12]	*d* = 0.64, *p* = .007^a^ 95CI [0.03, 0.18]

A greater proportion of commission errors occurred in the sexual condition than in the other conditions and men still made more errors than women in the sexual condition

^a^Indicates significance according to Bonferroni-Holm corrected threshold

### Reaction Times on Go-Trials

An ANOVA with the within-subjects factor *emotion* (neutral, positive, negative, sexual) and the between-subjects factor sex (male, female) revealed a significant large main effect of emotion, *F*(3, 219) = 16.59, *p* < .001, *η*_*p*_^2^ = 0.19, *ε*_GG_ = 0.61. Both the main effect of sex, *p* = .09, and the two-way-interaction, *p* = .35, were not significant.

**Fig. 3:**
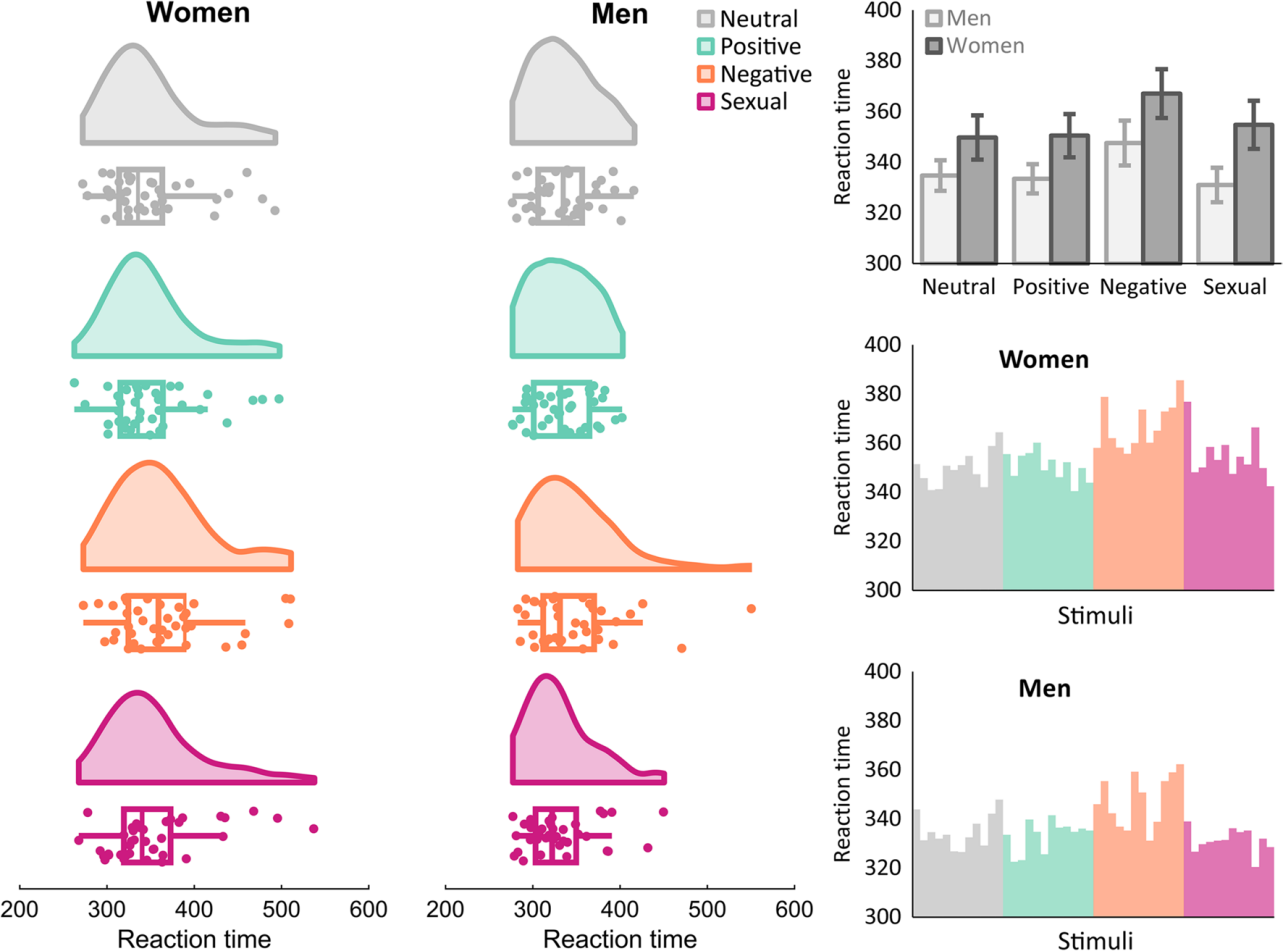
Response times to go stimuli. The left side shows raincloud plots including boxplots and the distribution of response times across participants. Mean values are depicted in the bar plot on the top right side. Despite descriptively longer response times in women, this difference was not significant. On the other hand, negative stimuli led to overall prolonged response times in comparison to the other emotion conditions, which could be interpreted as a sign of response inhibition effect. Error bars indicate standard errors of the mean. Mean response times for individual pictures used in the study can be obtained from the two bar plots on the mid and lower right side

Follow-up *t*-tests demonstrated that reaction times were slowed down in the negative condition in comparison to the neutral and the other emotional conditions, all *p*s < .001. No other comparisons were significant, *p*s > .64 (see Table 3 and Fig. 3).

**Table 3:** Statistics of response times in Go trials

	Men (*N* = 37)	Women (*N* = 38)	Overall (*N* = 75)	
	Mean (SD)	Mean (SD)	Mean (SD)	vs. Neutral
Neutral	335 (37)	350 (54)	342 (46)	–
Positive	333 (35)	351 (53)	342 (46)	*d* = 0.03, *p* = .778 95CI [−1.95, 2.59]
Negative	347 (54)	367 (59)	358 (57)	*d* = 0.56, *p* < .001^a^ 95CI [8.88, 21.26]
Sexual	331 (41)	355 (59)	343 (52)	*d* = 0.04, *p* < .746 95CI [−3.24, 5.22]

In Go trials participants responded slower in the negative condition than in the other conditions. No significant sex differences were observed

^a^Indicates significance according to Bonferroni-Holm corrected threshold

### Emotion Ratings

#### Valence

An ANOVA with the within-subjects factor *emotion* (neutral, positive, negative, sexual) and the between-subjects factor sex (male, female) revealed a significant large main effect of emotion, *F*(3, 207) = 257.52, *p* < .001, *η*_*p*_^2^ = 0.79, *ε*_GG_ = 0.75, and a significant medium-sized interaction of Emotion X Sex, *F*(3, 207) = 5.41, *p* = .004, *η*_*p*_^2^ = 0.07, *ε*_GG_ = 0.75. The main effect of *sex*, *p* = .24 was not significant.

For each group, follow-up *t*-tests were conducted and the significance threshold was Bonferroni-Holm corrected for six pairwise comparisons. Both men and women rated positive stimuli (men: 76.58 ± 11.14, women: 78.45 ± 13.05) as more positive than neutral stimuli (men: 59.92 ± 10.67, women: 58.50 ± 8.86), *p* < .001, and negative stimuli (men: 11.96 ± 14.64, women: 16.18 ± 16.44) as more negative than neutral stimuli, *p* < .001. However, only men rated sexual stimuli (men: 72.45 ± 19.49, women: 58.61 ± 21.55) as more positive than neutral stimuli, *p* = .001, while women rated neutral and sexual images equally in valence, *p* = .98. The rating of sexual images also differed significantly between groups, *p* = .006. There was no significant difference between sexual and positive images in men, *p* = .20, while positive images appeared more positive than sexual images to women, *p* < .001.

#### Arousal

An ANOVA with the within-subjects factor *emotion* (neutral, positive, negative, sexual) and the between-subjects factor *sex* (male, female) revealed a significant large main effect of *emotion*, *F*(3, 207) = 91.31, *p* < .001, *η*_*p*_^2^ = 0.57, *ε*_GG_ = 0.85. The main effect of sex, *p* = .343, and the two-way-interaction, *p* = .09, were not significant.

**Fig. 4:**
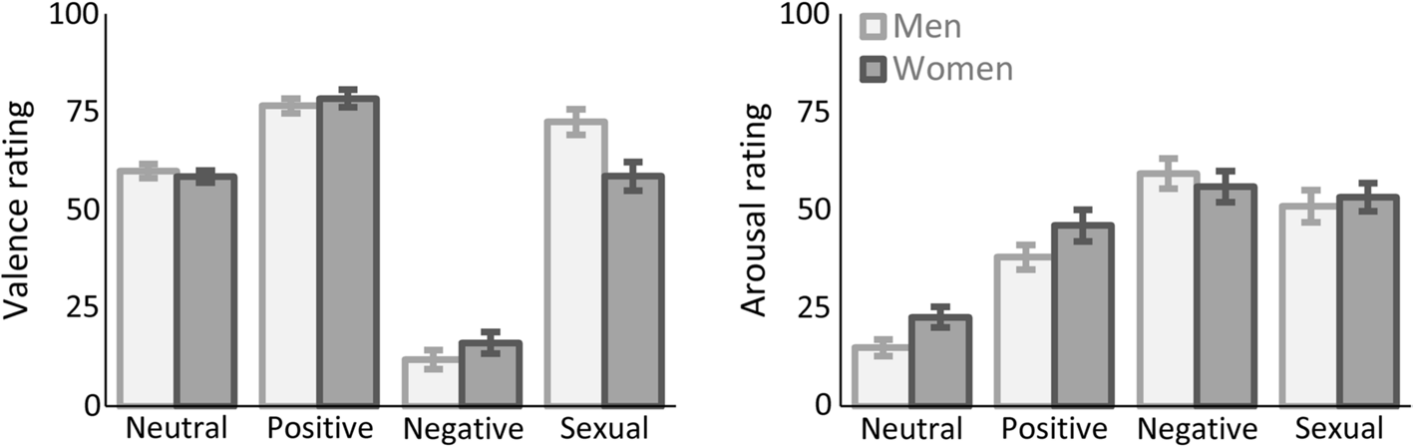
Mean valence and arousal ratings of emotional pictures. Zero indicates unpleasantness, resp. low arousal, while 100 indicates pleasantness, resp. high arousal. Positive and negative stimuli were significantly different from neutral stimuli in valence. Sexual stimuli were only rated more positive than neutral stimuli by men, and men rated them more positive than women. Overall, all emotional picture categories were rated as more arousing than the neutral category, while negative images were rated as most arousing. No sex differences emerged in arousal ratings. Error bars indicate standard errors of the mean.

Follow-up t-tests were conducted and the significance threshold was Bonferroni-Holm corrected for six pairwise comparisons. The emotion effect traced back to enhanced arousal of negative stimuli (57.61 ± 23.23), sexual stimuli (52.06 ± 22.96) and positive stimuli (42.03 ± 21.59), relative to neutral stimuli (18.78 ± 14.50), *p*s < .001. Also, both negative and sexual images were rated as more arousing than positive images, *p*s < .001, and negative images were rated as more arousing than sexual images, *p* = .026 (Fig. 4).

### Pupil Dilation

#### Go/No-Go Task

An ANOVA with the within-subject factors emotion (neutral, positive, negative, sexual) and time (0–1 s, 2–3 s, 3–4 s), Go/No-Go (go with response, nogo without response) as well as the between-subjects factor sex (male, female), resulted in significant main effects of emotion, *F*(3, 210) = 106.25, *p* < .001, *η*_*p*_^2^ = 0.60, *ε*_GG_ = 0.63, time, *F*(2, 140) = 12.91, *p* < .001, *η*_*p*_^2^ = 0.16, *ε*_GG_ = 0.84 and Go/No-Go, *F*(1, 70) = 131.82, *p* < .001, *η*_*p*_^2^ = 0.65. Moreover, there were significant interactions of Emotion X Time, *F*(6, 420) = 60.17, *p* < .001, *η*_*p*_^2^ = 0.46, *ε*_GG_ = 0.32, and Gonogo X Time, *F*(2, 140) = 142, *p* < .001, *η*_*p*_^2^ = 0.67, *ε*_GG_ = 0.77.

The pupil response to Go trials was enhanced relative to nogo trials in the first, *t*(71) = 4.44, *p* < .001, *d* = 0.23, 95CI [0.0058, 0.015], second, *t*(71) = 12.81, *p* < .001, *d* = 0.96, 95CI [0.077, 0.11], and third second of picture presentation, *t*(71) = 10.72, *p* < .001, *d* = 0.80, 95CI [0.052, 0.076]. That difference between go and nogo trials was more pronounced in the second second than before and after, *p*s < .001. Regarding the emotion effect, sexual stimuli evoked larger responses than all the other conditions with large effect sizes throughout picture presentation, while responses to negative stimuli were larger than positive at the beginning and larger than negative stimuli at the end of picture presentation. In detail, within the first second, sexual stimuli (0.074 ± 0.049) evoked larger pupil responses than neutral (0.048 ± 0.045) and all other conditions, *p*s < .001, *d*s > 0.93, and negative stimuli (0.052 ± 0.045) evoked larger responses than positive stimuli (0.047 ± 0.046), *t*(71) = 2.54, *p* = .013, *d* = 0.29, 95CI [0.00022, 0.00855]. The same pattern was observed in the second time interval with even larger effects for sexual stimuli (0.147 ± 0.113), *p* < .001, *d*s > 1.38, while in addition, pupil dilations to negative stimuli (0.055 ± 0.087) were enhanced relative to both positive (0.038 ± 0.089) and neutral stimuli (0.040 ± 0.091), *p* < .008, *d*s > 0.32. Finally, in the third second, negative stimuli (0.0049 ± 0.081) were enhanced relative to neutral stimuli (−0.012 ± 0.080), *t*(71) = 2.53, *p* = .014, *d* = 0.30, 95CI [0.0035, 0.0294], but not relative to positive stimuli (−0.0063 ± 0.084) anymore, *p* = .13. Still, sexual stimuli (0.123 ± 0.121) led to larger responses than all other stimuli, *p*s < .001, *d*s > 1.19.

#### Rating Phase

An ANOVA comprising the within-subject factors *emotion* (neutral, positive, negative, sexual) and *time* (0–1 s, 2–3 s, 3–4 s), as well as the between-subjects factor *sex* (male, female), resulted in large significant main effects of emotion, *F*(3, 195) = 70.41, *p* < .001, *η*_*p*_^2^ = 0.52, *ε*_GG_ = 0.89, and time, *F*(2, 130) = 88.39, *p* < .001, *η*_*p*_^2^ = 0.58, *ε*_GG_ = 0.68, and a significant interaction of Emotion X Time, *F*(6, 390) = 58.80, *p* < .001, *η*_*p*_^2^ = 0.48, *ε*_GG_ = 0.57. There was no significant impact of sex on pupil dilation.

**Fig. 5:**
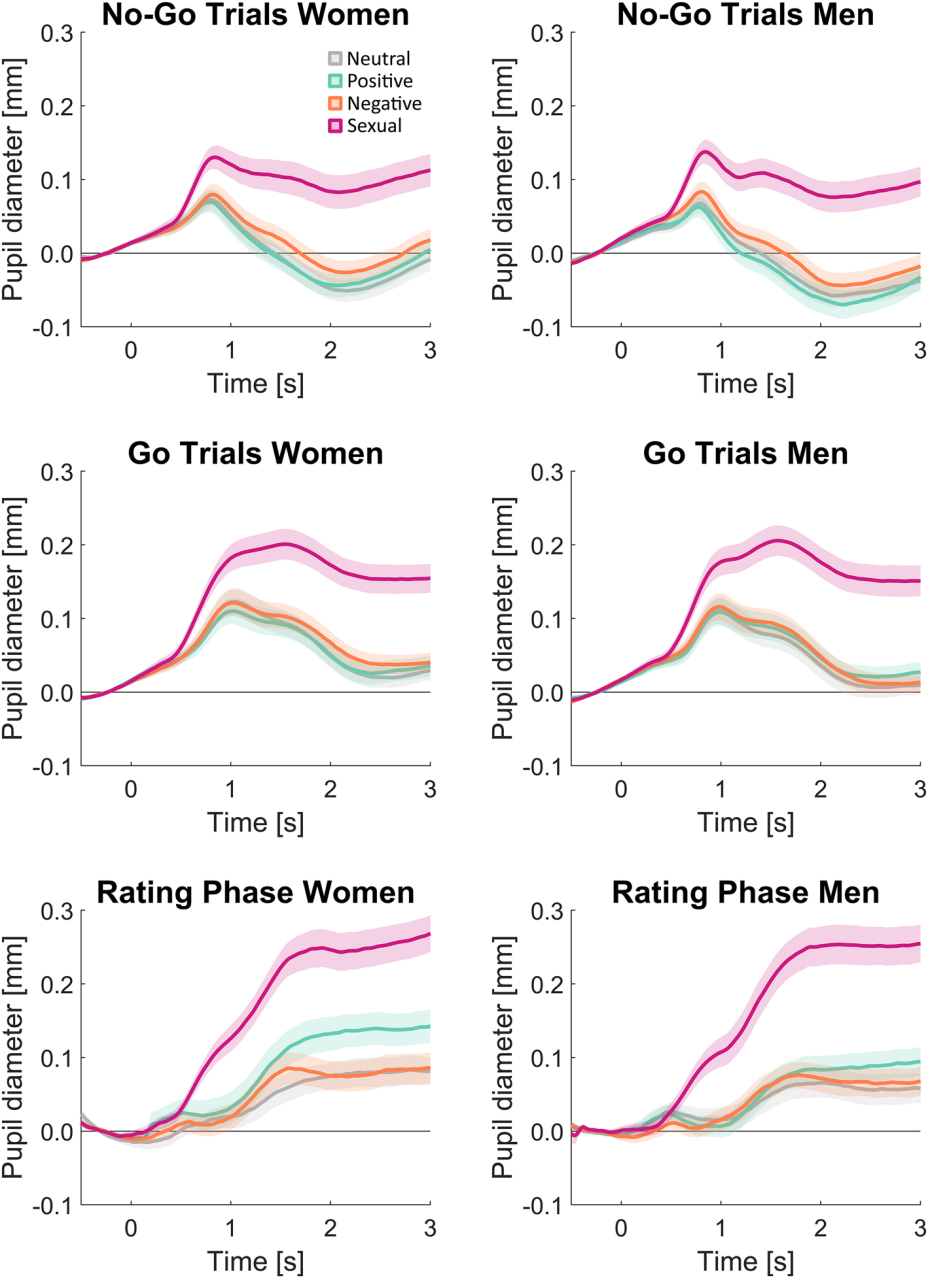
Pupil dilation in response to emotional pictures during the Go/No-Go task (top two rows) and the rating phase after the Go/No-Go task in which pictures were merely viewed passively (third row). Overall, no sex differences or interactions with sex were observed, but emotion effects were prevalent. Go trials evoked larger pupil dilations than No-Go trials, especially from second one to two. Sexual stimuli evoked large effect sizes relative to the other picture conditions throughout picture presentation. Negative stimuli evoked larger pupil dilations than positive stimuli in the first two seconds and larger than neutral picture in the last two seconds. In the rating phase, sexual stimuli still evoked larger pupil dilations than other conditions, while positive stimuli also evoked enhanced pupil dilation in the last second, but negative stimuli did not differ from neutral stimuli anymore. Shaded areas indicate standard errors of the mean

In order to resolve the significant interaction, separate ANOVAs were run for each time interval, which all resulted in significant main effects of emotion, *p*s < .001. For each time interval, the significance threshold was corrected for six pairwise comparisons. Within the first second, only sexual stimuli (0.04 ± 0.06) evoked larger pupil dilations than neutral images (0.01 ± 0.06), *t*(66) = 5.18, *p* < .001, *d* = 0.64, 95CI [0.02, 0.05]. This effect became larger within the second second (0.20 ± 0.13 vs. 0.05 ± 0.11), *t*(66) = 12.65, *p* < .001, *d* = 1.55, 95CI [0.13, 0.17], while positive images (0.07 ± 0.12) tended to be larger than neutral images but did not reach the Bonferroni corrected significance threshold (*α* = 0.017), *p* = .02. Within the third second of picture presentation, both sexual images (0.25 ± 0.15), *t*(66) = 12.51, *p* < .001, *d* = 1.53, 95CI [0.15, 0.21], and positive images (0.11 ± 0.12), *t*(66) = 3.98, *p* < .001, *d* = 0.49, 95CI [0.02, 0.07], evoked larger pupil dilations than neutral images (0.07 ± 0.12). Pupil dilation was also always larger for sexual images than for other emotions, *p*s < .003, and within the third second positive images evoked larger pupil dilation than negative images, *p* = .003. Negative images did not differ significantly from neutral images throughout picture presentation, *p*s > .19 (Fig. 5).

## Discussion

In the present study we examined the effects of positive, negative and sexual stimuli on response inhibition in women and men. Sexual stimuli substantially impaired the ability to withhold responses in the No-Go condition, revealing an increase in impulsivity in both women and men. However, the increase in impulsivity was significantly larger in men than in women. Although a trend for non-sexual positive stimuli emerged as well, this sex difference was specifically enhanced for sexual stimuli. On the other hand, no sex differences occurred for negative stimuli. Images of ill or injured people induced a similarly inhibiting effect in both men and women, as indicated by response times. While commission errors are considered the primary outcome of aGo/No-Go task, presumably indicating failed response inhibition or hyper-responsivity, response times may reflect processes such as vigilance or an approach tendency.

Different explanations have been suggested for emotional effects on response inhibition. One possibility is that emotional stimuli attract attention, facilitate visual processing and might thus facilitate or impair response inhibition, depending on whether the emotional stimulus is task relevant or not and how much cognitive resources are consumed by the emotional stimulus that are also necessary for response inhibition (Pessoa et al., 2012). This explanation also suggests that stimulus induced arousal is more relevant to the effects than valence. Following this framework, irrelevant sexual stimuli in the present study attracted attention and may also have consumed more central resources, presumably in prefrontal executive networks. This might have impaired the ability to inhibit a prepotent motor activity, which relies on a shared pool of resources (Moratti et al., 2004; Pessoa, 2009; Rubia et al., 2003; Shackman et al., 2009). As a result, response inhibition fails in the presence of sexual stimuli. However, within only an arousal based framework, we cannot explain why negative stimuli clearly did not lead to reduced response inhibition. Arousal ratings indicated that negative stimuli were even more arousing than sexual stimuli and pupil dilations during the go-nogo task were also increased relative to neutral and positive stimuli. Still, response inhibition was not reduced according to commission errors, but may even have been increased according to delayed response times in Go trials. Also, if sexual stimuli drew attention away from the main task, the question remains why reaction times were not delayed as in negative stimuli.

It seems irrefutable that emotions facilitate visual and attentional processes and we think these processes are also involved in the present findings (Compton, 2003; Schindler & Bublatzky, 2020; Schupp et al., 2006). However, this model alone does not seem to be sufficient to explain our results completely. We think an additional approach and avoidance model would best explain the current pattern of results (e.g., Gray, 1990). Sexual stimuli might induce a strong approach tendency that is harder to be inhibited than behavioral tendencies under neutral conditions. This leads to more commission errors or reduced reaction times. On the contrary, negative stimuli lead to an avoidance response that leads to fewer commission errors or prolonged reaction times. At this point, it is notable that the involvement of response inhibition in the Go/No-Go task is a matter of debate. The (hyper-) responsiveness in nogo trials cannot only be interpreted as a failure of response inhibition, but also as the result of a decision making process to respond or not to respond (Berkman et al., 2017; Veling et al., 2022). By this account, the value of responding is accumulated as well as the value of not responding and if the net value of responding crosses a certain threshold a response is initiated. Translated to the present results, sexual stimuli could be of higher value for men than for women (which seems to be supported by valence ratings) and/or committing errors could be less aversive to men (which seems to be supported by lower punishment sensitivity in men; Cross et al., 2011). In addition, sexual stimuli might also capture more of men’s attention or their value might be integrated in the decision making process more quickly, thus crossing a critical response threshold earlier. This explanation based on value accumulation and response threshold might be a sufficient and simpler explanation than traditional dual process theories of response and response inhibition. Wessel (2018) also questions the involvement of response inhibition in many go-nogo tasks, since a slow pace and the equal probability of go and nogo trials leads to a reduction in inhibition-related electrophysiological potentials. However, the frequency of Go trials and the instructions to respond very quickly should have established a certain pre-potency of the go response in the present study.

Numerous studies found that appetitive stimuli, such as food, drugs or desired sexual partners activate the dopaminergic mesolimbic reward system (Alonso-Alonso et al., 2015; Arias-Carrión et al., 2010; Stark et al., 2019). The activation of the nucleus accumbens as the terminal region of this system in response to food or sexual stimuli also predicted weight gain and sexual activity six months after brain activity was measured (Demos et al., 2012). Subliminally presented sexual cues evoke activity in the nucleus accumbens as well and dopamine regulates its activation as indicated by dopaminergic drug administration (Oei et al., 2012). In fact, dopamine systems seem to be most relevant for sexual excitation responses (Pfaus, 2009). The power and impact of the dopaminergic reward system on human behavior can also be seen in the life dominating consequences of drug abuse and addiction to which it very likely contributes (Wise & Koob, 2014). Similarly, men’s motivational approach systems in the brain may have been more engaged in the processing of sexual stimuli. The stronger the approach motivation, the more demanding the task of response withholding for an inhibitory control system.

The observed sex differences might trace back to deficient functioning of an inhibitory system, the increased approach motivation or both. It should be noted that the framework of independent approach and inhibition factors has also been incorporated more specifically for sexual interest and arousal in the dual control model of sexual response by Janssen and Bancroft (2007). According to this model, sexual arousal is the result of state and trait sexual excitation and inhibition. Sexual inhibition can be related to worries about the consequences of sexual behavior, but also about the failure of sexual performance. Interestingly, Carpenter et al. (2008) report lower self-reported sexual excitation and higher sexual inhibition in women than in men, which might also account for men’s enhanced behavioral responsiveness in the present study. Likewise, women’s sexual excitability does not seem to follow classical linear models of sexual arousal in an equal way as men’s sexual excitability (Giraldi et al, 2015). Several models for the unfolding of sexual arousal have been proposed, one of them assuming a linear increase from initial excitement and desire to a climax and return to a relaxed state (Masters & Johnson, 1966). Another model, which women seem to endorse more than men, assumes that sexual activity often starts with non-sexual desires (such as emotional closeness) and sexual desire then follows with the built up of sexual arousal (Basson, 2000). This suggests that explicit sexual images may play a less important role in sexual arousal compared with men.

The present findings of reduced response inhibition in the presence of sexual stimuli may help to explain why both women and men sometimes engage in risky sexual behavior or infidelity despite conflicting long-term goals. Unlike previous experiments, we found a modulating effect of sexual stimuli in both women and men. One reason for this may be that pictures were presented simultaneously with nogo stimuli here, thus the overlap between the emotional reaction and the response selection may have been stronger. In addition, sexual images were more explicit and possibly more arousing in the present study, resembling contemporary pornographic material. Finally, the role of cultural differences between a previous Taiwanese sample and a German sample cannot be excluded (Yu et al., 2012). Despite an effect in women and men, men were even more likely to fail in response inhibition. Valence ratings do suggest that men found sexual stimuli more pleasant than women and we did not find differences in the neutral condition or self-reported impulsivity. A former study also indicates no sex differences on the Barratt Impulsiveness Scale (Stanford et al., 2009) and executive response inhibition in neutral non-social contexts usually does not differ between men and women (Cross et al., 2011). While this may support the hypothesis of increased approach motivation (instead of decreased response inhibition), the picture may not be as clear as it seems. Women showed very similar arousal ratings and pupil dilations, suggesting that emotional responses—and possibly approach motivation—may have been comparable. Moreover, a recent study found better performance in women in a neutral go-nogo task (Sjoberg & Cole, 2018) and women and men indicated similar self-reported sexual disinhibition under experimentally induced sexual arousal (Imhoff & Schmidt, 2014). However, the present main findings and an overview of the literature seem to be more in accordance with the assumption that response inhibition is similar under neutral circumstances, but women outperform men in social and especially sexual contexts.

According to Bjorklund and Kipp (1996) women have evolved domain-specific inhibitory mechanisms that help to conceal sexual interest in potential partners. As women do invest more in individual offspring than men due to pregnancy and limited number of gametes, they have to be more cautious in the selection of partners. In this framework, it may also be possible that the approach motivation and response inhibition are similar, but response inhibition is more effective in certain domains. Behavioral inhibition may also vary with the phase of the menstrual cycle with more inhibition in the fertile late follicular phase (Hosseini-Kamkar & Morton, 2014). Here, according to self-report, women’s menstrual phases were distributed equally across the menstrual cycle, suggesting no major biases in the present data. Enhanced responsiveness in the presence of sexual stimuli in males may also indirectly reflect evolved mating strategies (Buss & Schmitt, 2019). Since female reproductive value is assumed to be closely linked to physical cues, men seem to have an especially high preference for physical attractiveness in a potential partner. A possible evolved mediating mechanism for enhanced responsivity may be sex differences in brain structure that facilitate connectivity between perception and action areas (Ingalhalikar et al., 2014).

As expected, images showing injured or mutilated individuals led to increased inhibition as indicated by response times. However, there were no indicators of increased responsiveness in men. In general, the results are in line with previous findings of faster response times to threatening negative pictures but slowed down response times to mutilation images (Buodo et al, 2017). The images may trigger defensive reactions such as fear, disgust or even more complex and social feelings like compassion. It has been suggested that blood-related stimuli initiate a defensive freezing response (Hagenaars et al., 2012). According to the present results, violence in men does not occur from a lack of similar defensive responses (see also Campbell, 2006, for a discussion of psychological moderators). But, again, it should be kept in mind that real life situations are more complex and significantly differ from simplified behavioral tasks as the present one. Future studies might test the effect of threatening or anger provoking stimuli on response inhibition.

A further potential follow-up direction would be to investigate how response inhibition under sexual stimulation can be improved. A previous study found that the training of response inhibition leads to a reevaluation of erotic stimuli (Driscoll et al., 2018; Ferrey et al., 2012). This may also dampen an approach motivation in those with excessive inhibition deficits. Recently, mindfulness training has been shown to be effective in improving response inhibition in a socioemotional context (Quaglia et al., 2019).

One limitation of the present study might be the focus on visual sexual stimuli. Although sex differences in sexual arousal are not completely understood, sexual arousal in women may be more influenced by contextual factors than sexual arousal in men (Rupp & Wallen, 2008). These contextual factors might be less accessible in visual cues. There is also evidence that the correlation between subjective sexual arousal and objectively measured genital responses is lower in women than in men (Chivers et al., 2004). Also, men’s pupil dilation is more specifically increased in response to subjectively preferred sexual stimuli than in women. While heterosexual men show an increase for female stimuli and homosexual men show an increase for male stimuli, women did not show a significant difference between male and female stimuli, as a recent meta-analysis concludes (Attard-Johnson et al., 2021). However, one could argue that sexual arousal and sexual inhibition are two sides of the same medal and sexual arousal without context in the presence of superficial visual cues might already be a form of sexual disinhibition. Still, in the absence of further studies using different modalities of sexual stimuli, the current results might not be generalized to sexual arousal irrespective of less complex visual stimulation. In addition, one should be cautious in generalizing the present results to everyday examples of sex differences in sexual behavior. Although we think that an enhanced behavioral responsiveness to sexual stimuli might play a role in impulsive decision making, real life behavior is much more complex and several other situational and psychological factors will have an influence that might have an additional or even overwriting effect.

In conclusion, the present findings of increased impulsivity in the presence of sexual stimuli are in concordance with an increased approach motivation and/or reduced inhibitory capacities in men. The increased approach motivation might trace back to a more positive value of visual sexual cues to men, the capture of attention by sexual cues or a more efficient integration of sexual stimuli into response selection processes. These findings of (hyper-) responsiveness in the presence of sexual stimuli might in part help to explain risky or transgressing behavior in real life, but future studies will still have to examine their correlation.

## Data Availability

We report how we determined our sample size, all data exclusions and manipulations. This study was not pre-registered. Data are available at https://figshare.com/s/ec17115bb656ee16dc17 (to be replaced by doi).
